# Vaccination in Health Care Workers Should be Following in our Country Now?!

**DOI:** 10.5812/ircmj.17145

**Published:** 2013-12-05

**Authors:** Seyed Moayed Alavian

**Affiliations:** 1 Baqiyatallah Research Center for Gastroenterology and Liver Diseases, Baqiyatallh University of Medical Sciences, Tehran, IR Iran

**Keywords:** Hepatitis A, Vaccination, Health Personnel, Prevention

Dear Editor,

I read with interest the published article by Bayani et al. (1) in your esteemed journal recently. The topic is important for health policy makers to find better view regarding epidemiology of hepatitis A virus (HAV) infection in the community. HAV is an enteric transmitted infection and it is the most common of causes of acute viral hepatitis in Iran and the world (2-4). This HAV is a transmitted via fecal-oral route virus through person to person contact and contaminated water and food and the mode transmission is slightly different in various parts of the world according to its prevalence in each region. The reported data from Iranian different groups showed significant differences across the regions (2-5). Different in sewage disposal with secondary contamination of drinking water and foods are the reason for this difference.

Bayani et al. (1) reported the significantly increase in sero-prevalence with age, 77.4% for 30-39 years and 85.3% for more than 40 years. less prevalence in lower age has confirmed in other studies in general populations from different parts of Iran (5). Considering that the disease has more severe course as age increases, improvement of standard hygiene and prevention strategies are recommended. Furthermore, vaccination may play a significant role in the occupational health policy to protect the susceptible health care workers population in the future (6). Karimi Elizee et al. (7) reported higher rate of anti-HAV antibody in thalassemia patients in comparison with hemophilia group and they concluded that other risk for transmission in hospitalization and receiving more blood may be affective in this differences. I would like to refer to Bayani et al. (1) that they are right and other risk factors for acquiring in health care workers should more be attend by health policy makers. Around to 30% of the health workers have been seronegative and are still at risk of HA infection development. I would like to sensitize the nurses and other health care workers to be more aware and careful in approach to acute viral hepatitis patients in medical wards.

Finally I would like to mention that adding to educational approach for health care workers and more attending to health precautions during work, vaccination of non-immune individuals against HAV infection in this high risk group is wisdom strategy now (8, 9). Vaccination against HAV can prevent from morbidity and mortality due to acute fulminant hepatitis and it can prevent HAV will not spread in the society, and this will decrease the probability of HAV outbreak (10). It is important to follow other studies to find the cost-benefit of screening before vaccination in future.
